# Prevalence and Associated Factors of Emotional and Behavioural Difficulties during COVID-19 Pandemic in Children with Neurodevelopmental Disorders

**DOI:** 10.3390/children7090128

**Published:** 2020-09-04

**Authors:** Jacqueline Nonweiler, Fiona Rattray, Jennifer Baulcomb, Francesca Happé, Michael Absoud

**Affiliations:** 1 Department of Children’s Neurosciences, Evelina London Children’s Hospital, Guy’s and St Thomas’ NHS Foundation Trust, London SE1 7EH, UK; jacqueline.nonweiler@kcl.ac.uk (J.N.); Jennifer.Baulcomb@gstt.nhs.uk (J.B.); 2 Social, Genetic and Developmental Psychiatry Centre, Institute of Psychiatry, Psychology and Neuroscience, King’s College London, London SE5 8AH, UK; fiona.rattray@kcl.ac.uk (F.R.); francesca.happe@kcl.ac.uk (F.H.); 3 Department of Women & Children’s Health, King’s College, London SE1 7EH, UK

**Keywords:** autism spectrum disorder, attention-deficit/hyperactivity disorder, behavioural neuroscience, mental health, COVID-19, pandemic, paediatric neurology

## Abstract

Children and young people (CYP) with neurodevelopmental disorders (NDDs) may be particularly vulnerable to adverse mental health effects due to the COVID-19 pandemic. We conducted a cross-sectional U.K. parent-reported study from 2nd April–2nd June 2020, using the Strengths and Difficulties Questionnaire. CYP with NDDs (*n* = 371), compared to neurotypical controls, had a higher prevalence of emotional symptoms (42% vs. 15%) and conduct problems (28% vs. 9%), and fewer prosocial behaviours (54% vs. 22%). All groups had worse emotional symptoms than pre-COVID groups, and those with attention-deficit/hyperactivity disorder showed inflated conduct problems, while those with autism spectrum disorder exhibited decreased prosocial behaviours. Females with ASD had higher emotional symptoms compared to males. CYP with NDDs, and those without, showed higher levels of parent-reported mental health problems than comparable cohorts pre-COVID-19.

## 1. Introduction

Children and young people (CYP) worldwide may be particularly vulnerable to the adverse mental health effects of lockdown, school closures, and physical distancing measures due to the COVID-19 pandemic [1,2]. Compared to neurotypical CYP, those with neurodevelopmental disorders (NDDs) such as autism spectrum disorder (ASD) and attention-deficit/hyperactivity disorder (ADHD) may be more vulnerable still [3]. These CYP often struggle with changes in routine and restrictions to activity. It is imperative to understand the early mental health impacts across these common disorders to inform reasonable adjustments and interventions. Hence, we examined the prevalence of emotional and behavioural problems and associated factors for these symptoms in children with NDDs during the early stages of the COVID-19 pandemic.

## 2. Methods

We conducted a cross-sectional United Kingdom parent-reported study from 2nd April (10 days after the start of UK lockdown) to 2nd June 2020. We recruited a population-based convenience sample of children via social media and/or email lists disseminated via NDD charities and schools (King’s College London Research Ethics Committee: LRU-19/20-15033). Using the Strengths and Difficulties Questionnaire, a standardized outcome measure used widely in observational studies with 25 items across 5 behavioural domains [4], we compared our data to previously published norms and a UK mental health cohort chosen due to similar characteristics [5,6]. We report descriptors; subscale mean scores (emotional, conduct, hyperactivity, peer, and prosocial); between-groups pairwise results after adjustment for multiple comparisons, age, sex, and developmental level (general linear model multivariate procedure; IBM^®^ SPSS v26.0, Armonk, NY, USA); and effect sizes.

## 3. Results

Data for 453 children aged 4–15 years were analyzed (320 males; 70.6%). The neurodevelopmental diagnostic categories reported were ASD (*n* = 106), ADHD (*n* = 183), and comorbid ASD + ADHD (*n* = 82). CYP had an additional reported neurological diagnosis in 12% (*n* = 3 cerebral palsy; 9 epilepsy; 10 genetic; 11 tic disorder; and 12 developmental co-ordination disorder). Neurotypical CYP (*n* = 82) showed worse scores than norms on emotional symptoms and prosocial behaviour sub-scales (Table 1) [5]. When compared to a pre-COVID-19 mental health cohort, our clinical samples’ scores reflect notably worse mental health in emotional symptoms, hyperactivity, and prosocial behaviour (Supplementary Table S1 and Figures S1–S6). Compared to neurotypical controls, children with NDDs had a higher prevalence of emotional symptoms (42% vs. 15%; χ^2^ = 21.0, *p* < 0.001), higher conduct problems (28% vs. 9%; χ^2^ = 13.2, *p* < 0.001), and lower prosocial behaviours (54% vs. 22%; χ^2^ = 22.5, *p* < 0.001). The emotional subscale was elevated across clinical groups (effect size 0.7–0.98); those with ADHD showed inflated conduct problems (effect size 1.12–1.17), and those with ASD exhibited decreased prosocial behaviours (effect size 0.97–1.06). Females with ASD had considerably higher emotional symptoms compared to males (mean (SD) = 7.2 (2.5) vs. 5.2 (2.8); t = 4.7, *p* < 0.001).

## 4. Discussion

These data suggest a high prevalence of emotional and behavioural difficulties in CYP, particularly with NDDs, during the early period of the COVID-19 pandemic. The comorbid ASD + ADHD group was particularly impaired with respect to conduct and prosocial behaviours. Females with ASD were particularly vulnerable to increased emotional symptoms compared to males. CYP in all groups showed more emotional symptoms than comparable pre-COVID-19 cohorts. The advantages of our method of recruitment include allowing data to be collected in a short time and mitigating barriers to community-based data collection imposed by the pandemic. Limitations include cross-sectional data, and self-selected parent reports, which are potentially prone to bias. In addition, we did not have data on whether subjects with NDDs were under clinical care in the ‘’COVID19 lockdown period’’, be it using telerehabilitation tools or in person. These data require replication in other studies, including those employing random sampling methods.

Children’s vulnerability to mental health changes, especially for those with NDDs, should be considered when integrating them back into schools and daily life. Whilst managing their own transitions back into new ways of working, clinicians, parents, and teachers can likely expect increased mental health needs from CYP with NDDs that will require immediate support. A focus on wellbeing approaches with individualized multi-disciplinary transition planning, seeking psychological resources, and increasing respite services for families may help ease reintegration. In addition, CYP with NDDs often have other neurological conditions that require management and are often impacted by mental health difficulties [1]. The risk of second waves of COVID-19, and the reduction of social care, respite services, and after school clubs pose further threats for families who have children with NDDs. The pandemic hence risks widening inequalities for children, particularly in vulnerable groups. As such, this presents an opportunity to redress the imbalance and support children and families’ wellbeing as we emerge into the ‘’new normal’’ world.

## Figures and Tables

**Table 1:** Strengths and Difficulties Questionnaire data for neurodevelopmental disorders (NDD) clinical groups and controls during lockdown, and UK (pre-COVID-19) norms (*n* = 10,298) [4].

SDQ Subscale	Diagnostic Category	Mean (SD)	95% CI	Effect Size (Hedges’ g) vs. Control (95% CI)	Pairwise Comparisons for Study Groups ^†^ and Summary *t*-Test for Controls vs. UK Norms
Emotional symptoms (0–10)	ASD	5.9 (2.8)	5.4–6.4	0.98 (0.67, 1.28)	ASD; ADHD; ASD + ADHD > control (*p* < 0.01)
	ADHD	5.1 (2.8)	4.7–5.5	0.70 (0.43, 0.97)	
	ASD + ADHD	5.5 (2.8)	4.9–6.2	0.84 (0.52, 1.16)	
	Control	3.1 (2.9)	2.5–3.8		Controls > norms *t* = 3.9, *p* < 0.001
	UK norms	1.9 (2.0)			
Conduct problems (0–10)	ASD	3.1 (2.2)	2.7–3.6	0.52 (0.22, 0.81)	ADHD; ASD + ADHD > ASD > control (*p* < 0.01)
	ADHD	4.6 (2.2)	4.3–4.9	1.17 (0.80, 1.45)	
	ASD + ADHD	4.5 (2.2)	4.0–5.0	1.12 (0.79, 1.44)	
	Control	2.0 (2.3)	1.5–2.5		Control vs. norms—*t* = 1.5, *p* = 0.1
	UK norms	1.6 (1.7)			
Hyperactivity/inattention (0–10)	ASD	6.8 (2.0)	6.4–7.2	1.39 (1.06, 1.70)	ADHD; ASD + ADHD > ASD > control (*p* < 0.01)
	ADHD	9.2 (2.0)	4.3–4.9	2.56 (2.21, 2.89)	
	ASD + ADHD	8.9 (2.0)	8.4–9.3	2.42 (2.01, 2.81)	
	Control	4.0 (2.1)	3.6–4.5		Control vs. norms—t = 1.8, *p* = 0.07
	UK norms	3.5 (2.6)			
Peer relationships (0–10)	ASD	6.0 (2.2)	5.6–6.4	1.80 (1.45, 2.14)	ASD; ADHD; ASD + ADHD > control (*p* < 0.01)
	ADHD	3.9 (2.3)	3.6–4.2	0.87 (0.60, 1.14)	
	ASD + ADHD	5.6 (2.2)	5.2–6.1	1.64 (1.28, 1.99)	
	Control	1.9 (2.3)	1.4–2.4		Control vs. norms—*t* = 1.7, *p* = 0.09
	UK norms	1.5 (1.7)			
Prosocial behaviour (0–10)	ASD	4.3 (2.4)	3.9–4.8	−1.06 (−1.37, −0.75)	ASD; ASD + ADHD < ADHD, control (*p* < 0.01) ADHD < control (*p* = 0.04)
	ADHD	6.1 (2.4)	5.7–6.4	−0.37 (−0.63, −0.11)	
	ASD + ADHD	4.6 (2.4)	4.0–5.1	−0.97 (−1.29, −0.65)	
	Control	7.0 (2.5)	6.4–7.5		Control < norms for *t* = 5.91, *p* < 0.001
	UK norms	8.6 (1.6)			

^†^ Bonferroni corrections for multiple comparisons, and corrected for developmental level, age, and sex. ASD = Autism Spectrum Disorder (M:F = 67:39; mean age = 10.4 (3.1) years); ADHD = Attention Deficit Hyperactivity Disorder (M:F 142:41; mean age = 10.4 (2.6) years); ASD + ADHD = comorbid ASD and ADHD (M:F = 62:20; mean age = 11.0 (2.7) years); controls = neurotypical children (M:F 49:33; mean age = 9.6 (3.5) years); Hedges’ g: measure of effect size where values of 0.20, 0.50, and 0.80, equivalent to effect sizes as small, medium, or large; ns = non-significant difference; *t* = *t*-test.

## Supplementary Materials

The following are available online at https://www.mdpi.com/2227-9067/7/9/128/s1, Figure S1: SDQ total scores, Figure S2: SDQ emotional symptoms, Figure S3: SDQ conduct problems, Figure S4: SDQ hyperactivity, Figure S5: SDQ peer problems, Figure S6: SDQ prosocial behaviour, Table S1: Comparison of strengths and difficulties questionnaire data for COVID-19 lockdown clinical group (*n* = 371) versus a UK clinical (pre-COVID-19) clinical mental health sample (*n* = 6846).
