# Supplementary material for: Prevalence and Associated Factors of Emotional and Behavioural Difficulties during COVID-19 Pandemic in Children with Neurodevelopmental Disorders
Source: Children (Basel). 2020 Sep 4;7(9):128. doi: 10.3390/children7090128 (PMC7552706; doi:10.3390/children7090128)

Supplementary Table and Figures

Table S1: Comparison of Strengths and Difficulties Questionnaire data for COVID-19 lockdown clinical group (n=371) versus a UK clinical (pre-COVID-19) clinical mental health sample (n=6846)[6]

SDQ Subscale	Study sample†	Mean (SD)	Effect size (Hedges' g [95% CI])	Summary t-test
Emotional symptoms (score 0-10)	Nonweiler et al	5.5 (2.9)	0.28 (0.17-0.38)	Current > SLaM t = 5.2, p<0.001
	Fernandez de la Cruz et al	4.7 (2.8)		
Conduct problems (score 0-10)	Nonweiler et al	4.2 (2.3)	0.11 (0-0.21)	Current > SLaM t = 2.0, p=0.04
	Fernandez de la Cruz et al	3.9 (2.6)		
Hyperactivity/ inattention (score 0-10)	Nonweiler et al	8.4 (2.0)	0.78 (0.68-0.89)	Current > SLaM t = 20.30, p<0.001
	Fernandez de la Cruz et al	6.2 (2.9)		
Peer relationships (score 0-10)	Nonweiler et al	4.9 (2.5)	0.55 (0.44-0.65)	Current > SLaM t = 10.20, p<0.001
	Fernandez de la Cruz et al	3.6 (2.4)		
Prosocial behaviour (score 0-10)	Nonweiler et al	5.2 (2.6)	-0.51 (-0.62,-0.41)	Current < SLaM t = -9.51, p<0.001
	Fernandez de la Cruz et al	6.5 (2.6)		

† Current study; ASD, ADHD and ASD+ADHD diagnostic groups collapsed into one clinical group; age range 4-15 years; n = 371
A clinical sample from the South London and Maudsley (SLaM) NHS Foundation Trust, serving a population of approximately 1.2 million residents; mean age=11.2 (SD=3.8); n = 6846

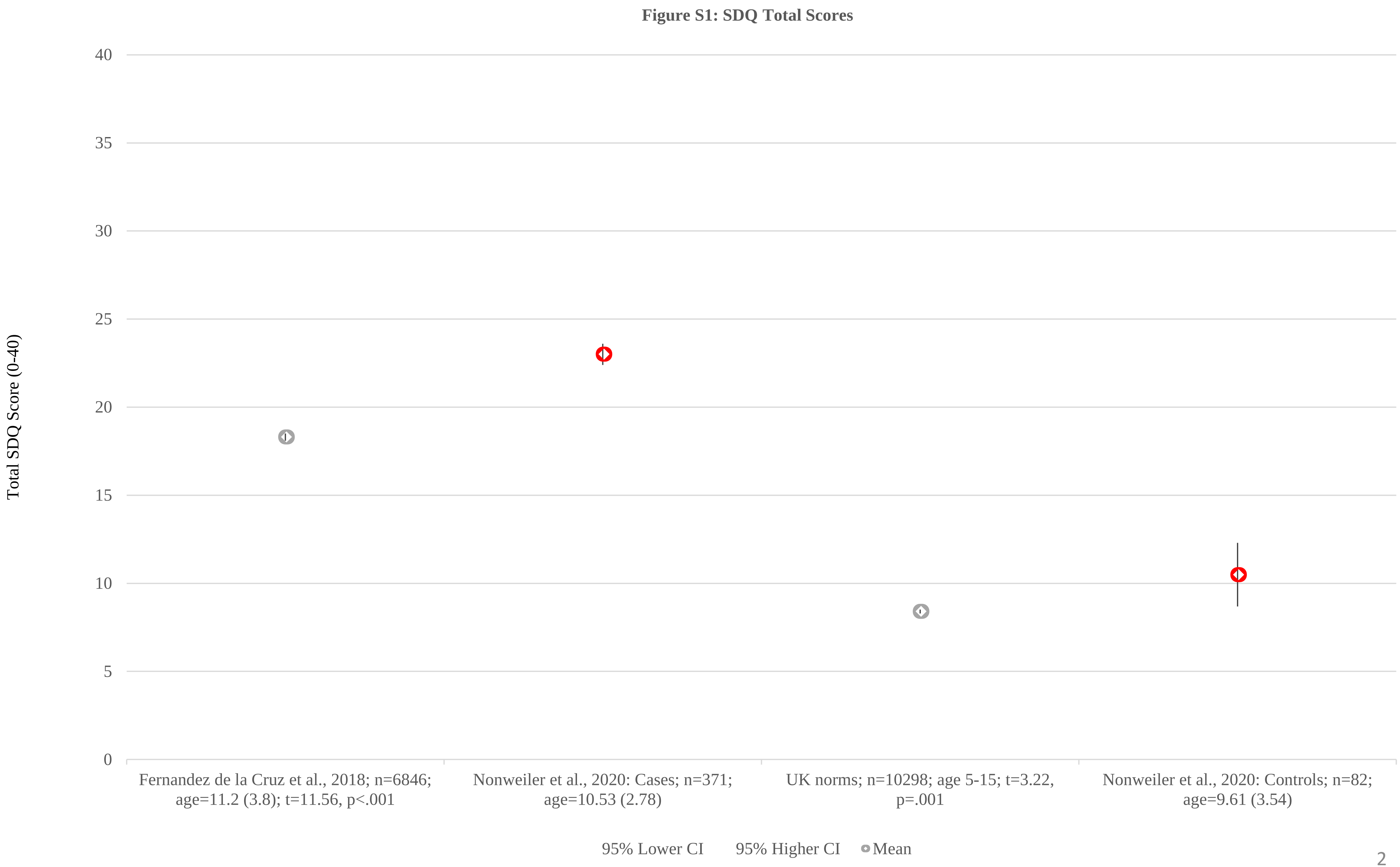
Figure S1: SDQ Total Scores
40
35
30
25
20
15
10
5
0
Total SDQ Score (0-40)
Fernandez de la Cruz et al., 2018; n=6846; age=11.2 (3.8); t=11.56, p<.001
Nonweiler et al., 2020: Cases; n=371; age=10.53 (2.78)
UK norms; n=10298; age 5-15; t=3.22, p=.001
Nonweiler et al., 2020: Controls; n=82; age=9.61 (3.54)
95% Lower CI
95% Higher CI
Mean

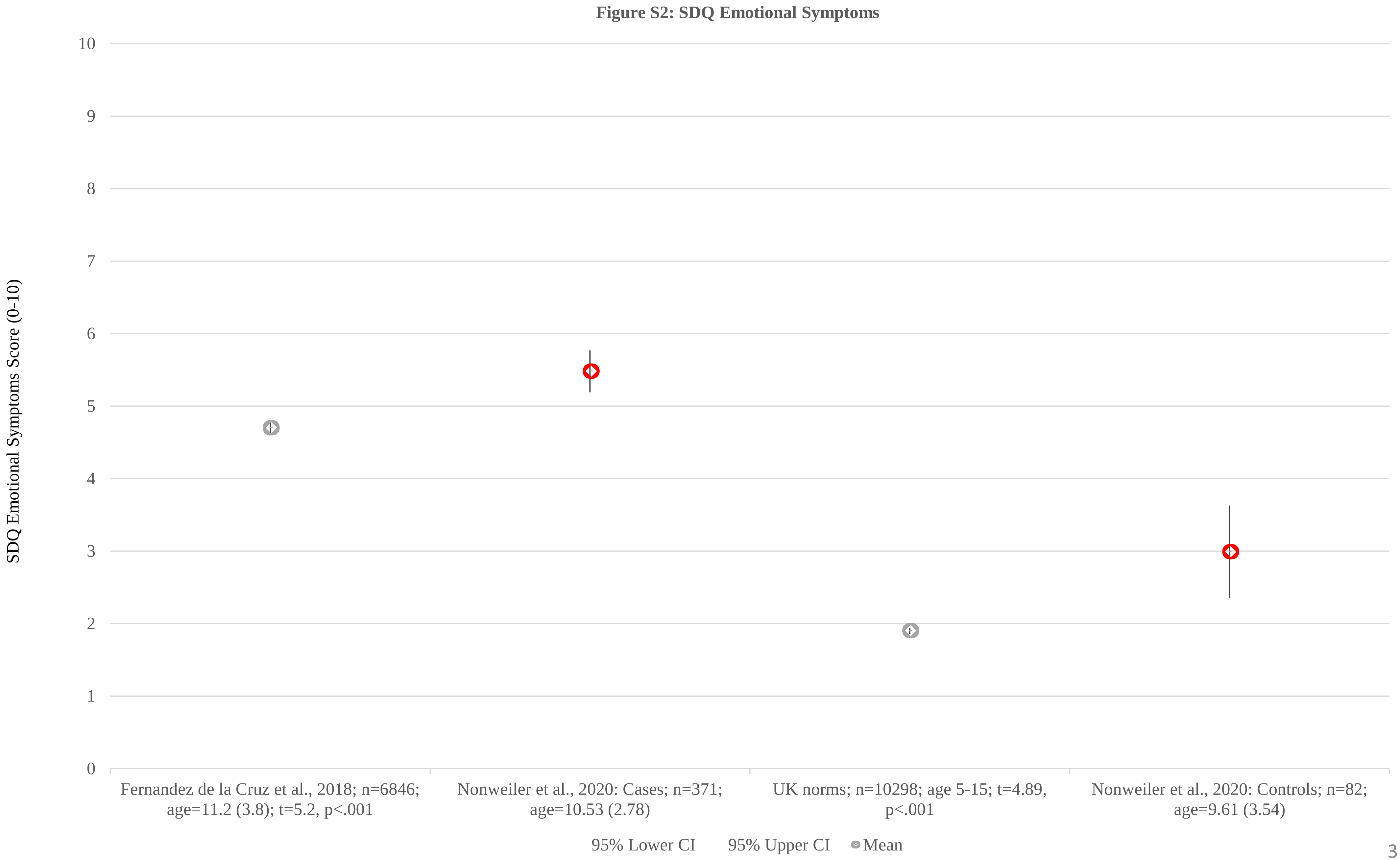
Figure S2: SDQ Emotional Symptoms
SDQ Emotional Symptoms Score (0-10)
10
9
8
7
6
5
4
3
2
1
0
Fernandez de la Cruz et al., 2018; n=6846; age=11.2 (3.8); t=5.2, p<.001
Nonweiler et al., 2020: Cases; n=371; age=10.53 (2.78)
UK norms; n=10298; age 5-15; t=4.89, p<.001
Nonweiler et al., 2020: Controls; n=82; age=9.61 (3.54)
95% Lower CI
95% Upper CI
Mean

Figure S3: SDQ Conduct Problems

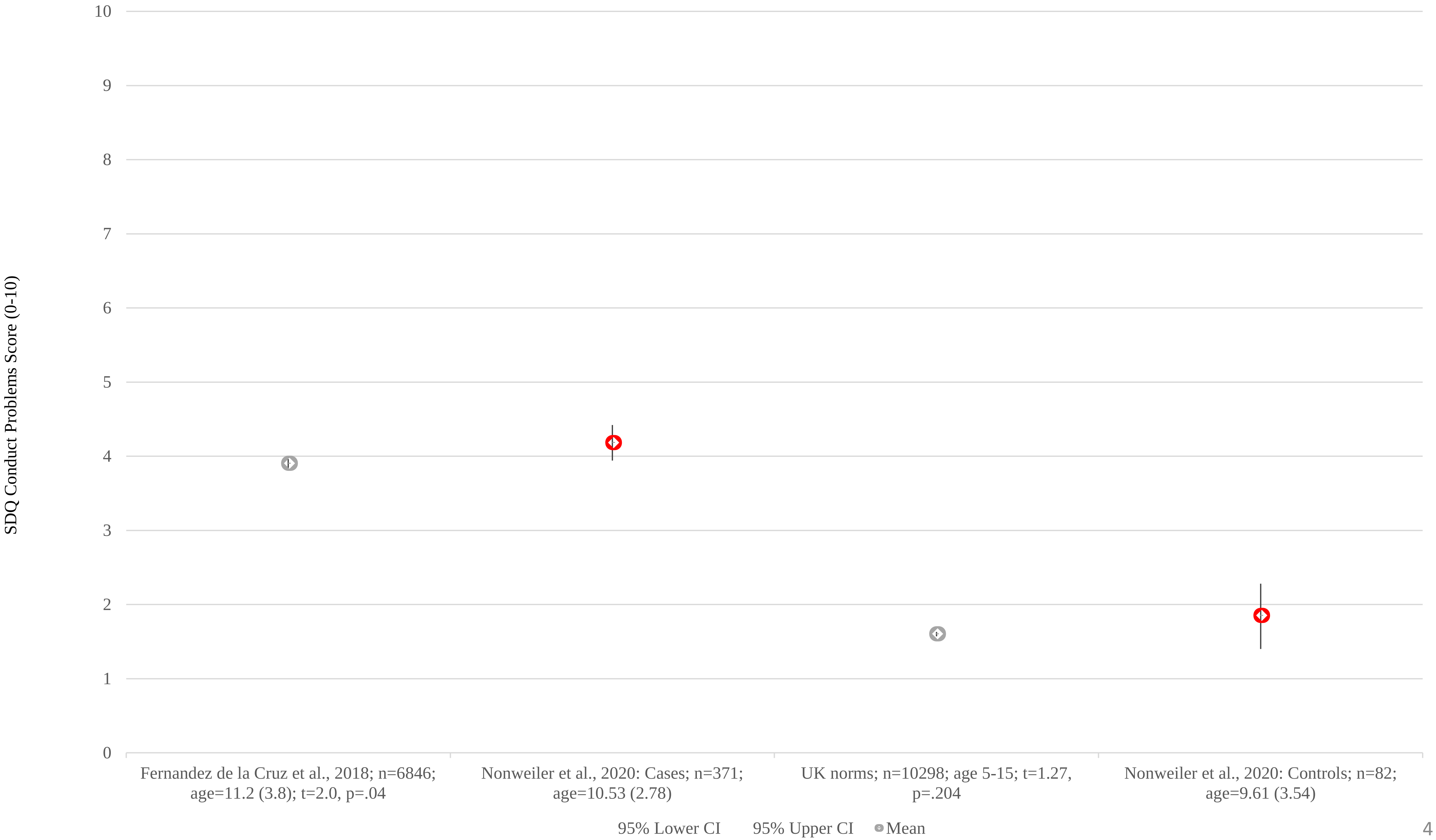

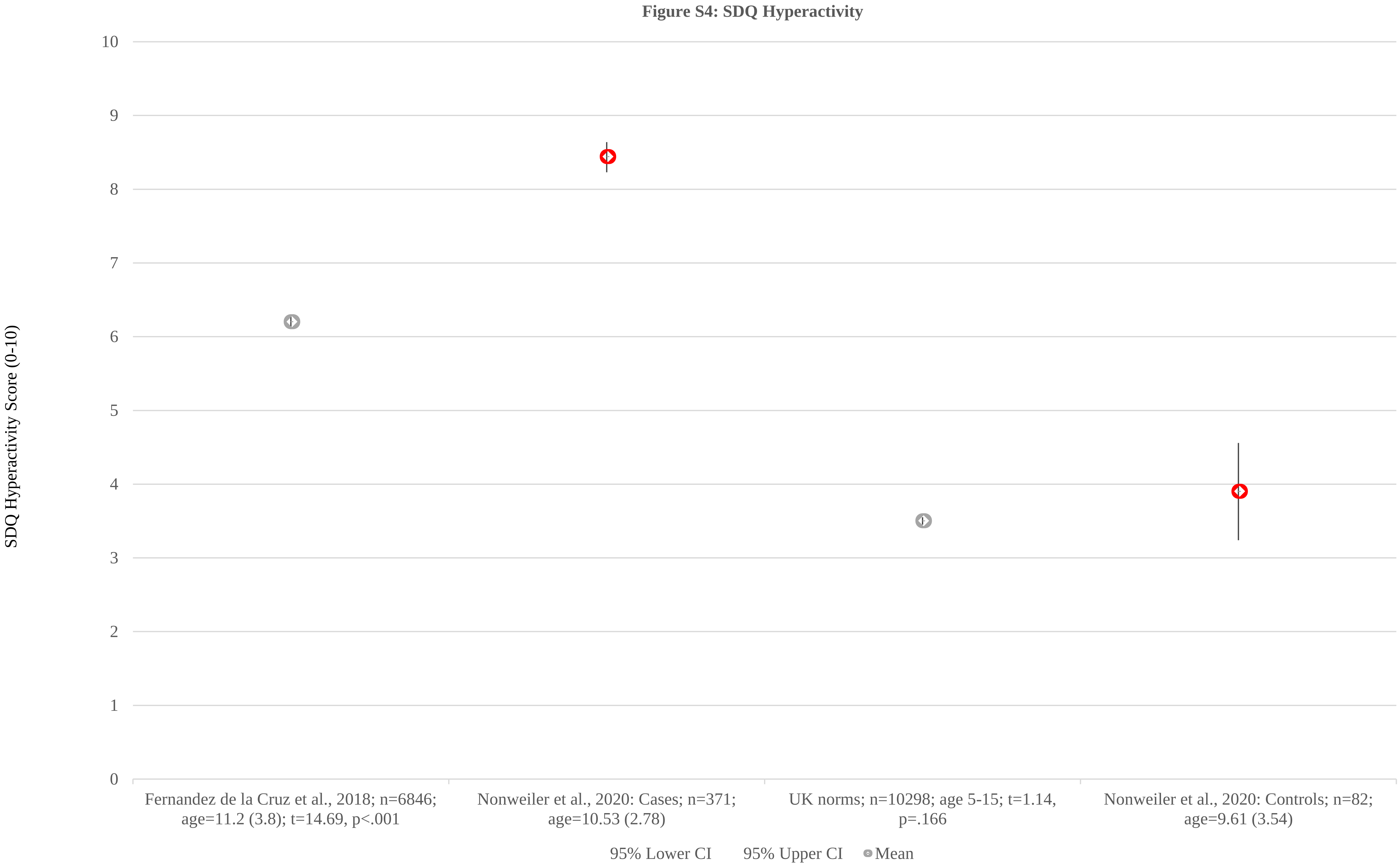
Figure S4: SDQ Hyperactivity
SDQ Hyperactivity Score (0-10)
10
9
8
7
6
5
4
3
2
1
0
Fernandez de la Cruz et al., 2018; n=6846; age=11.2 (3.8); t=14.69, p<.001
Nonweiler et al., 2020: Cases; n=371; age=10.53 (2.78)
UK norms; n=10298; age 5-15; t=1.14, p=.166
Nonweiler et al., 2020: Controls; n=82; age=9.61 (3.54)
95% Lower CI
95% Upper CI
Mean

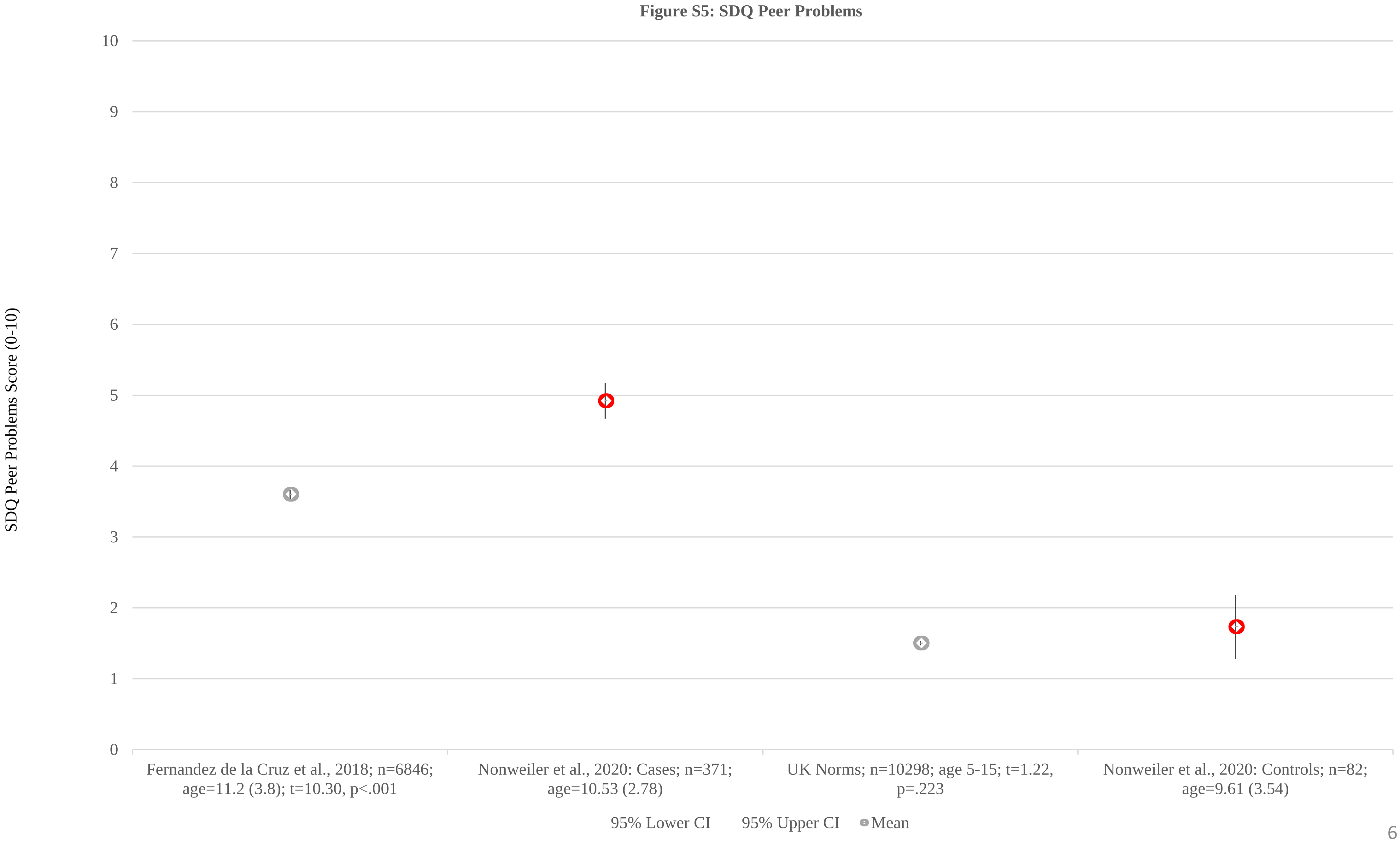
Figure S5: SDQ Peer Problems
SDQ Peer Problems Score (0-10)
10
9
8
7
6
5
4
3
2
1
0
Fernandez de la Cruz et al., 2018; n=6846; age=11.2 (3.8); t=10.30, p<.001
Nonweiler et al., 2020: Cases; n=371; age=10.53 (2.78)
UK Norms; n=10298; age 5-15; t=1.22, p=.223
Nonweiler et al., 2020: Controls; n=82; age=9.61 (3.54)
95% Lower CI
95% Upper CI
Mean

Figure S6: SDQ Prosocial Behaviour

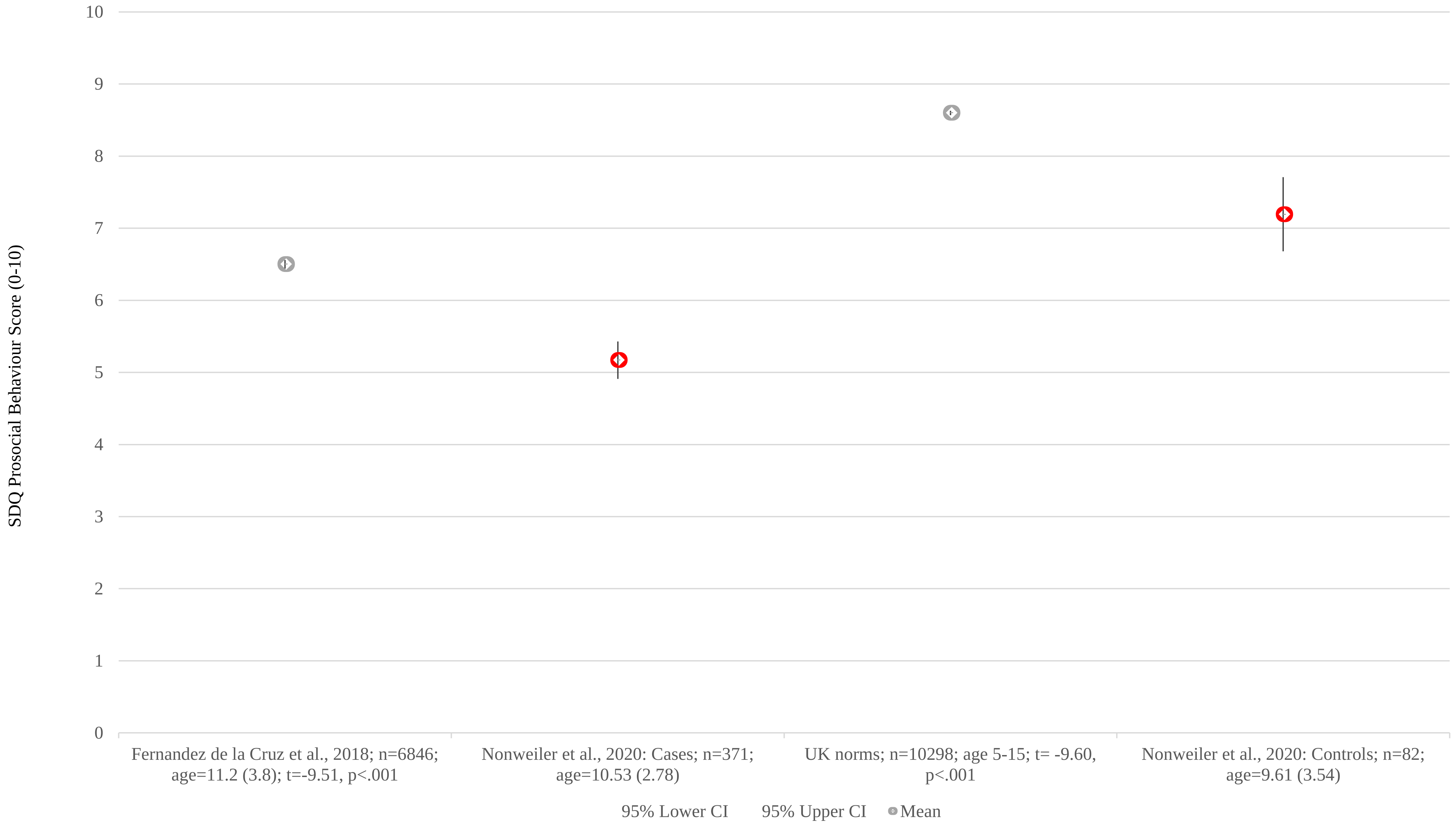

Supplement: Supplementary file 1 [file children-07-00128-s001.pdf]
